# Supplementary material for: Effects of Trichoderma harzianum combined with Phanerochaete chrysosporium on lignin degradation and humification during chicken manure and rice husk composting
Source: Front Microbiol. 2025 Feb 28;16:1515931. doi: 10.3389/fmicb.2025.1515931 (PMC11906335; doi:10.3389/fmicb.2025.1515931)
Supplement: Supplementary file 1 [file Data_Sheet_1.zip › 6.Function/CKF_BFHF/FunGuild/l2/metastats/Bryophyte_Parasite.CKF_BFHF.metastats.pdf]

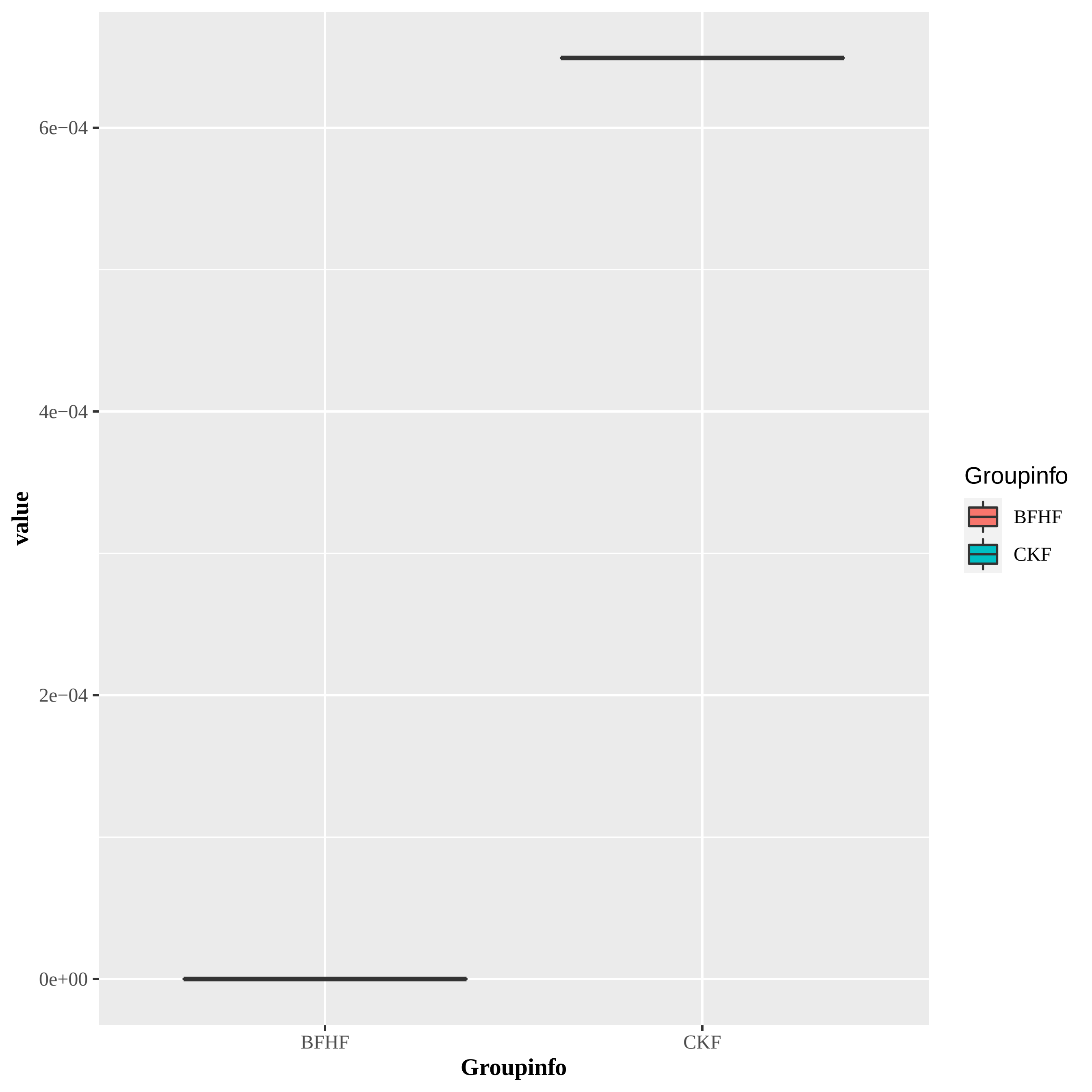

6e−04
4e−04
2e−04
0e+00
value
BFHF
CKF
Groupinfo
Groupinfo
BFHF
CKF
